# Effects of Lead Exposure on 1573 Male Workers’ Sex Hormones in China

**DOI:** 10.3390/toxics13050415

**Published:** 2025-05-21

**Authors:** Ping Wang, Zhiling Wu, Ju Li, Yue Li, Xuefeng Wang, Mengya Ma, Wenkai Wei, Yijun Wang, Yi Liu, Yi Sun, Ling Tao, Yanyan Yang, Ziyuan Zhou, Jingchao Ren, Jia Cao, Guanghui Zhang

**Affiliations:** 1 Department of Labor Health, School of Public Health, Shanxi Medical University, Taiyuan 030001, China; wangping2182025@163.com; 2 Department of Environmental Health, College of Preventive Medicine, Third Military Medical University (Army Medical University), Chongqing 400038, China; wzl2023@tmmu.edu.cn (Z.W.); ziyuanzhou@tmmu.edu.cn (Z.Z.); 3 School of Inspection, Xinyang Vocational and Technical College, Xinyang 464000, China; liju@126.com (J.L.); taoling2023@126.com (L.T.); 4 Henan International Collaborative Laboratory for Health Effects and Intervention of Air Pollution, School of Public Health, Xinxiang Medical University, Xinxiang 453003, China; 13598630857@163.com (Y.L.); 18137452386@163.com (X.W.); 15639371833@163.com (M.M.); 15738662823@163.com (W.W.); 19712567097@163.com (Y.W.); 19561980108@163.com (Y.L.); 18438392379@163.com (Y.S.); 5 Xinyang Center for Disease Control and Prevention, Xinyang 453003, China; jerry12799@163.com; 6 School of Public Health, Chongqing Medical University, Chongqing 400038, China; 103250@cqmu.edu.cn; 7 Key Lab of Medical Protection for Electromagnetic Radiation, Ministry of Education of China, Institute of Toxicology, College of Preventive Medicine, Third Military Medical University (Army Medical University), Chongqing 400038, China

**Keywords:** blood lead levels, mediating effects, luteinizing hormone, testosterone, sex hormones

## Abstract

Lead (Pb) is recognized as an environmental pollutant with male reproductive toxicity, but its effects on sex hormones remain unclear. This study investigated the relationship between male blood lead levels (BLLs) and the sex hormones of luteinizing hormone (LH), follicle-stimulating hormone (FSH), and prolactin (PRL), as well as testosterone (T), estrogen (E2), and progesterone (PROG). Observational and experimental data from 1573 Pb-exposed workers (712 had also been surveyed in the previous year) and 35 Pb-poisoned patients (before and after Pb chelation therapy) were analyzed. Results from a cross-sectional study showed a nonlinear relationship between BLLs and LH/FSH, and a linear relationship between BLLs and serum T. After Pb chelation therapy, the BLLs in patients decreased from 61.7 to 36.3 (μg/dL), serum T and FSH decreased significantly (*p* < 0.001), and serum LH also decreased but without a significant change, while PRL and PROG increased significantly (*p* < 0.01). The data indicate that Pb may disturb male sex hormones by including LH, T, and FSH, and this needs further research.

## 1. Introduction

Lead (Pb) is a toxic heavy metal that is widely present in the environment and constitutes one of the major risk factors for public health and the global burden of disease (GBD) [1]. Although Pb exposure has dramatically declined over the past two decades due to the ban on its use in gasoline, the GBD statistics estimate that approximately 900,000 individuals worldwide still die each year as a result of Pb exposure [2]. Furthermore, it is worth noting that Pb pollution is still increasing in developing countries [3]. Ample evidence indicates that high levels of Pb exposure can have toxic effects on human health, affecting the neurological, behavioral, immune, renal, hepatic, and digestive systems, and can also lead to behavioral abnormalities and reproductive dysfunction in men [4,5]. To date, an estimated 413 million children have blood lead levels (BLLs) exceeding 10 µg/dL (U.S. Childhood Pb Poisoning Standard (10 µg/dL)) [6]. In addition to adverse neurological effects, the impact of Pb on male reproductive health is equally concerning.

The effects of environmental pollution, particularly Pb pollution, have garnered significant public attention due to the observed decline in both the quantity and quality of human semen over the past few decades [7]. Evidence from previous epidemiological studies, including our report, sufficiently confirms the adverse effects of Pb on male reproductive health [8,9]. In our prior study, we identified an inverse association between Pb exposure and semen quality, even at low exposure levels (blood Pb concentration less than 10 μg/dL) [9]. However, the underlying mechanisms of Pb toxicity remain incompletely understood. Pb is suspected to induce toxicity in the male reproductive and nervous systems by disrupting the hypothalamic–pituitary axis [10]. The sex hormones, including follicle-stimulating hormone (FSH), luteinizing hormone (LH), progesterone (PROG), testosterone (T), estrogen (E2), and prolactin (PRL), are regulated by the hypothalamic–pituitary–testis (HPT) axis, which plays a crucial role in male reproductive function. Investigating the relationship between Pb exposure and sex hormone levels is essential for elucidating the mechanisms underlying Pb-induced reproductive toxicity.

The relationship between Pb exposure and male sex hormone levels remains controversial. Among the studies conducted since 2000, the three most recent investigations [11,12,13] reported that Pb exposure induced elevated levels of LH and FSH, while four other recent studies [14,15,16,17] indicated that Pb exposure decreased the levels of LH and FSH. Additionally, a 2020 review found no association between the sex hormones FSH, LH, and testosterone (T) and Pb exposure in occupational workers; however, the authors noted the necessity for larger, higher-quality studies [18]. Furthermore, all environmental investigations are subject to confounding factors, including mixed exposures to other metals, which can introduce interference and bias. Similar to a recent report, no significant association was found between serum FSH concentrations and blood Pb levels (BLLs); however, the volunteers were also exposed to mercury (Hg) [19]. Another study observed a positive correlation between serum testosterone and BLLs in 869 American men exposed to cadmium (Cd) [20]. In conclusion, previous studies have primarily focused on a limited number of sex hormones in each investigation, leading to inconsistent results across both human and animal populations. This inconsistency is illustrated in two seminal reviews: one review of animal studies concluded that Pb exposure impairs the hypothalamic–pituitary axis [21], while another review of human investigations found no significant association between Pb exposure and serum hormone levels [18]. In occupational studies, subjects are primarily exposed to Pb in occupational settings, thereby minimizing confounding factors from other metals present in the environment. Given the established relationship between luteinizing hormone (LH) and testosterone (T) regulation via the hypothalamic–pituitary–testicular (HPT) axis, it is crucial to consider the potential impact of Pb exposure on LH and T levels. Therefore, further investigation is warranted to determine whether LH mediates the effects of Pb exposure on serum testosterone levels.

Considering the aforementioned factors, occupational epidemiological surveys and experimental epidemiological studies were conducted to further elucidate the relationship between Pb exposure and sex hormones at two levels: the secretion of follicle-stimulating hormone (FSH), luteinizing hormone (LH), and prolactin (PRL) by the pituitary gland and the secretion of testosterone (T), estradiol (E2), and progesterone (PROG) from the testes and renal capsule. In a cross-sectional survey, 1573 male workers from a Pb–acid battery factory were recruited, with 768 of them had also been surveyed in the previous year for sex hormone levels from the previous year, excluding 56 workers who were undergoing oral Pb-removal treatment. Eventually, 712 workers were tested once in each of the two years. Additionally, we enrolled 35 male occupational workers diagnosed with Pb poisoning who were receiving Pb chelation therapy, aiming to evaluate alterations in serum sex hormone profiles through longitudinal monitoring before and after treatment administration. All participants provided written informed consent prior to participation. To the best of our knowledge, this Pb-exposed population represents the largest occupational sample ever studied regarding the effects of Pb exposure on sex hormones. Furthermore, we evaluated dose–response relationships using restricted cubic spline (RCS) models. Finally, we analyzed the mediating effects between blood Pb levels, serum LH levels, and serum T levels. This study is, to our knowledge, the first to include combined levels of both pituitary and testicular hormones in an observational study, potentially providing new insights into the relationship between Pb exposure and androgens in the hypothalamic–pituitary–testicular (HPT) axis.

## 2. Materials and Methods

### 2.1. Study Population

**Figure 1:**
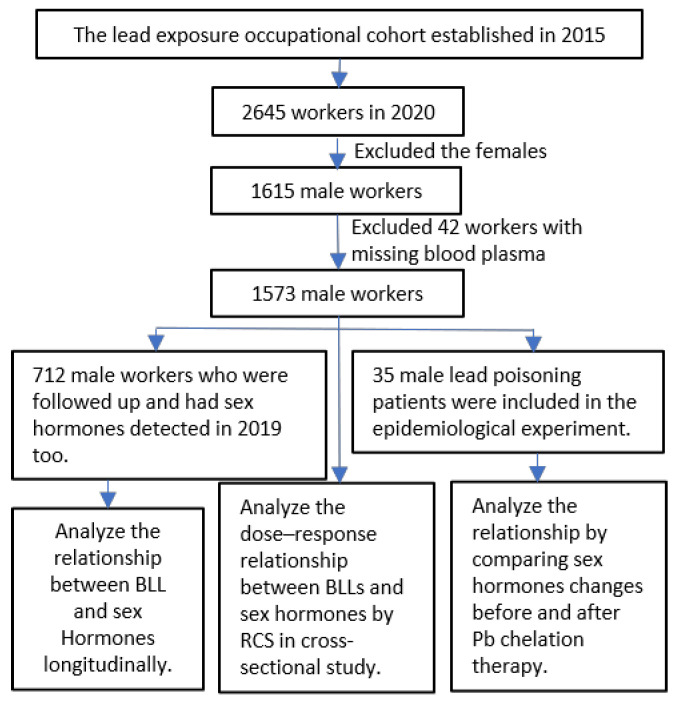
A flow chart displaying sample selection from the Pb–acid battery plant.

To investigate the impact of Pb exposure on public health in China, we established an occupational cohort at a Pb–acid battery factory in Henan Province in 2015, as illustrated in Figure 1. This cohort recruited over 2000 workers and included annual assessments comprising questionnaires, physical examinations, and blood samples. In a cross-sectional study conducted in 2020, we included 1573 male workers from the occupational cohort, of whom 712 were followed up in 2019 to assess their sex hormone levels. These male workers were subsequently included in the repeated cross-sectional study. The inclusion criteria were as follows: ① occupationally exposed populations refer to individuals engaged in Pb battery manufacturing or other operations involving Pb; specifically, this includes male workers who have been continuously exposed to Pb for a duration of one year or more while involved in the manufacture of Pb storage batteries or related activities; ② age range: 18–60 years old; ③ health status assessment, which included a physical examination upon entry and a pre-study clinical evaluation, revealing no significant diseases; additionally, the blood Pb test results were consistent with occupational exposure; and ④ informed consent: participation in this study required the voluntary signing of an informed consent form and cooperation in the completion of the study process. The exclusion criteria were as follows: individuals diagnosed with influenza, infections, or tumors were excluded from the study. In this epidemiological pilot study, we recruited 35 male patients with Pb poisoning who underwent a two-week Pb chelation therapy. Upon admission, the patients received a slow intravenous infusion of calcium sodium ethylenediaminetetraacetate (1.0 g of calcium sodium ethylenediaminetetraacetate in 250 mL of 5% dextrose solution). The intravenous administration of calcium ethylenediaminetetraacetate sodium salt was conducted over three consecutive days, with treatment ceasing after four days. Additionally, the patients were provided with reduced glutathione, Chandan injection, multivitamins, and other adjuvant therapies. This treatment regimen was repeated during the second week, and plasma samples were collected before and after the treatment.

After obtaining informed consent, the volunteers completed a questionnaire that included basic demographic information, their medical history, protective measures, smoking and alcohol consumption habits, lifestyle choices, and dietary habits. Additionally, their height, weight, and other relevant indicators were measured. Blood samples were collected from an elbow vein, immediately centrifuged at 1000 rpm for 10 min, and subsequently stored at −80 °C. The study was conducted in accordance with the Declaration of Helsinki (revised in 2013).

### 2.2. Experimental Design

To detect blood Pb levels (BLLs) in workers, heparin-anticoagulated whole blood was analyzed following the standard ”WS/T 174-1999” [22]: Chinese Graphite Furnace Atomic Absorption Spectrometry Method for the Determination of Pb and Cadmium in Blood. An Analyst 800 Atomic Absorption Spectrometer (Perkin Elmer, Thane, India) was employed, operating at an analytical wavelength of 283.3 nm, with a spectral passband of 0.7 nm and a lamp current of 10 mA. The temperature of the graphite furnace was controlled by an optical temperature controller, and data were recorded using the peak area integral method. Quality control was maintained through blank tests, parallel sample determinations, and standard addition recovery tests during the measurement of quality control samples. According to WS/T 174-1999, the limits of detection (LOD) and quantification (LOQ) of this method were 0.8 μg/dL and 2.5 μg/dL, respectively (calculated using the standard deviation of the blank sample). All measurements adhered to the standard values specified in the certificate, and the study was conducted by qualified technicians from the Xinxiang Occupational Disease Prevention and Control Hospital.

### 2.3. Hormone Measurement

Plasma samples anticoagulated with EDTA from workers exposed to Pb were utilized for the measurement of sex hormones. The analysis was conducted using a Roche e602 automatic electrochemiluminescence instrument (Cobas 8000 e 602, Basel, Switzerland), along with the appropriate supporting reagents. Hormone levels, including FSH, LH, PROG, T, E2, and PRL, were assessed in accordance with the manufacturer’s instructions. All hormone measurements were subjected to professional clinical training and quality control to ensure accuracy and reliability.

### 2.4. Statistical Analysis

Statistical analyses were performed using SPSS version 22.0 (IBM Corporation, New York, NY, USA) and SAS software, version 9.4, (SAS Institute Inc., Cary, NC, USA). The large samples of blood Pb levels (BLL) and sex hormones were approximately normally distributed and are expressed as the mean ± standard deviation (SD). Abnormal extremes exceeding the mean ± 2 SD were excluded from the analysis. The dose–response relationship between BLLs and sex hormones was assessed using restricted cubic spline (RCS) analysis in SAS software (version 9.4), as previously described [23]. The effect of BLLs on sex hormones was analyzed using multiple linear regression, adjusting for confounders such as age, body mass index (BMI), smoking, and alcohol consumption, based on the different BLL levels classified by RCS curves. Paired-sample *t*-tests and Wilcoxon signed-rank tests (based on results from paired-sample *t*-tests; Wilcoxon signed-rank tests are for reference only) were employed to compare the differences in blood Pb levels (BLLs) and sex hormone levels among workers monitored between 2019 and 2020. Additionally, we mapped the distribution of six sex hormones and blood Pb levels in 712 occupationally Pb-exposed men during this period using R (Version 4.3.1; R Core Team, Vienna, Austria 2023), along with R Studio (RStudio, Inc., Boston, MA, USA) and the ggplot2, haven, and gridExtra packages. Pearson correlation analysis was utilized to examine the relationship between sex hormones and variations in BLLs. Generalized linear regression was applied to analyze the effect of BLLs on sex hormones, adjusting for baseline data from 2019 and considering factors such as age, body mass index (BMI), smoking, and alcohol consumption. The primary reason for missing data in the two-year research group was the exclusion of extreme values for BLLs and sex hormone levels. Specifically, one sample in 2019 and three samples in 2020 exhibited prolactin (PRL) values exceeding 100 ng/mL, two samples in 2020 had estradiol (E2) values surpassing 1000 pg/mL, one sample in 2020 showed luteinizing hormone (LH) values greater than 40 mlU/mL, and one sample in 2020 had progesterone (PROG) values exceeding 15 ng/mL; all of these outliers were removed. For data from patients before and after Pb chelation therapy, we employed paired-sample *t*-tests and Wilcoxon signed-rank tests to compare differences in sex hormones. A *p* value of less than 0.05 was considered statistically significant.

## 3. Results

### 3.1. Participant Characteristics, Blood Pb Levels, and Sex Hormones

In 2020, we recruited 1573 male workers from a Pb–acid battery factory in Henan Province, China, and repeated measurements with 712 of these individuals over the period from 2019 to 2020. Additionally, 35 male patients who had been treated for Pb poisoning were included in the study, both before and after their Pb chelation therapy. The data are summarized in Table 1. In the cross-sectional analysis, the mean age of participants was 36.77 years, and the mean body mass index was 24.43 kg/m^2^. Notably, 59% of the participants reported smoking cigarettes, while 54% consumed alcohol. The mean and median blood Pb levels (BLLs) were 24.96 μg/dL and 23.63 μg/dL, respectively, with BLL values ranging from 2.71 to 67.76 μg/dL. The mean levels of FSH, LH, PROG, T, E2, and PRL in this cross-sectional study were 6.15 mIU/mL, 4.16 mIU/mL, 0.53 ng/mL, 4.58 ng/mL, 72.91 pg/mL, and 5.78 ng/mL, respectively.

### 3.2. Dose–Response Relationship Between Blood Pb Levels and Hormones in the Cross-Sectional Study

**Figure 2:**
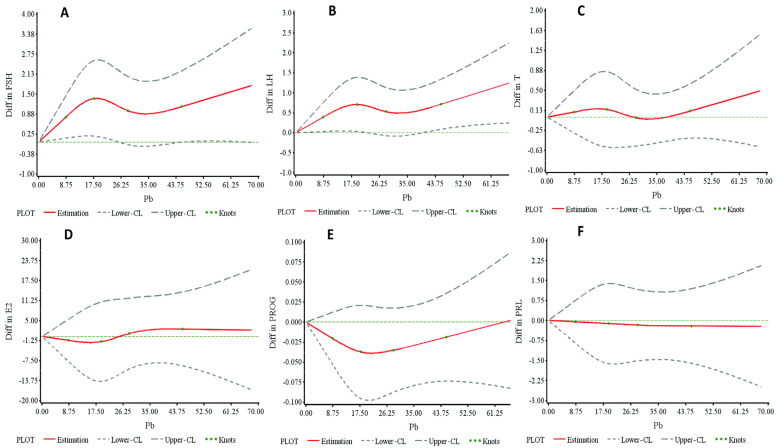
(**A**–**F**) The adjusted dose–response relationship between sex hormones and blood Pb levels was obtained through the restricted cubic spline (RCS) model in this cross-sectional study. The y-axis represents the difference (Diff) in sex hormone levels between individuals with any value of blood Pb level and those with the minimum blood Pb level after adjusting for age, body mass index (BMI), smoking habits, and drinking habits. The hormones analyzed include follicle-stimulating hormone (FSH), luteinizing hormone (LH), progesterone (PROG), testosterone (T), estrogen (E2), and prolactin (PRL).

Workers exposed to Pb were categorized into five groups based on blood Pb levels (BLLs) of 10, 20, 30, and 40 μg/dL. An analysis of variance (ANOVA) and the Kruskal–Wallis test were employed to assess the differences in sex hormone levels among these groups, as presented in Table S1. Testosterone (T) levels exhibited a tendency to increase with rising BLLs (*p*-trend = 0.021). Specifically, T levels in the highest BLL group were significantly elevated compared to those in the lowest BLL group (4.95 ± 2.26 vs. 4.28 ± 1.71, *p* = 0.006). Similarly, luteinizing hormone (LH) levels were also higher in the highest BLL group compared to the lowest (4.43 ± 2.22 vs. 3.85 ± 1.72, *p* = 0.018). Figure 2A–F illustrates the restricted cubic spline (RCS) analyses depicting the dose–response relationship between serum hormones and BLLs after adjusting for age, body mass index, smoking, and alcohol consumption. Figure 2A,B reveal analogous nonlinear relationships between BLLs and both follicle-stimulating hormone (FSH) and LH, with both hormones increasing as the BLL rises when the BLL exceeds 30.0 μg/dL. Figure 2C indicates a linear increase in T levels with the BLL. No significant associations were observed between BLLs and progesterone (PROG), estradiol (E2), or prolactin (PRL) (Figure 2C,D).

Table 2 illustrates the dose–response relationship between blood Pb levels (BLLs) and sex hormones, as determined by restricted cubic spline (RCS) curves. Serum luteinizing hormone (LH) levels showed a significant increase with rising BLLs when the levels exceeded 30.0 μg/dL (β = 0.027, 95% CI: 0.003–0.051, *p* = 0.028). Moreover, a linear increase in testosterone (T) levels was observed with rising BLLs for values greater than 28.09 μg/dL (*p* = 0.016). In contrast, for BLL levels below 20.5 μg/dL, serum follicle-stimulating hormone (FSH) levels also exhibited an upward trend with increasing BLLs (β = 0.069, *p* = 0.079).

**Figure 3:**
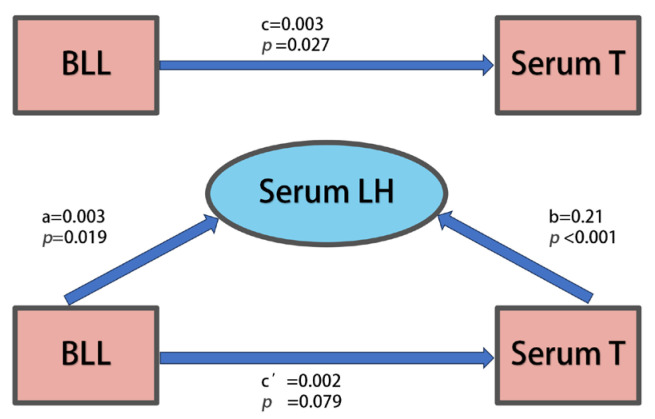
This study examines the mediation models involving the blood Pb level (BLL), serum testosterone (T), and serum luteinizing hormone (LH) within a cross-sectional framework. The mediating effects of serum LH were identified in the association between the BLL and serum T.

Figure 3 illustrates that serum luteinizing hormone (LH) mediates the relationship between blood Pb levels (BLLs) and serum testosterone (T). Notably, complete mediating effects of serum LH were observed when BLLs exceeded 30.0 μg/dL.

### 3.3. Relationships Between Blood Pb Levels and Hormones in Repeated Cross-Sectional Study

**Figure 4:**
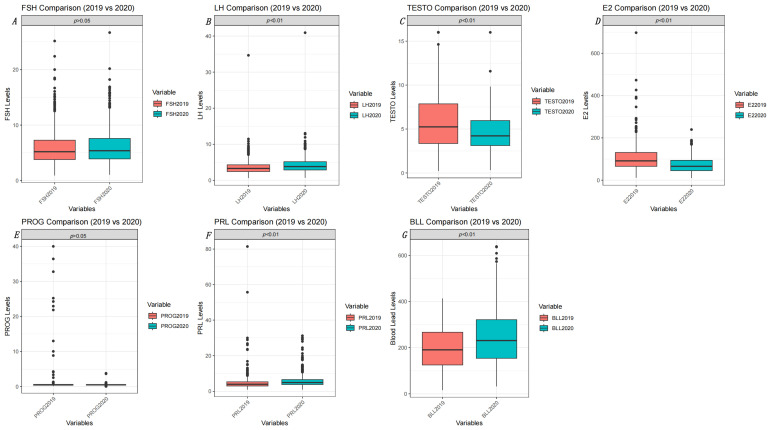
Serum six sex hormones and blood Pb levels in 2019 vs. 2020 in a repeated cross-sectional study: (**A**) FSH (*p* > 0.05); (**B**) LH (*p* < 0.01); (**C**) T (*p* < 0.01); (**D**) E2 (*p* < 0.01); (**E**) PROG (*p* > 0.05); (**F**) PRL (*p* < 0.01); (**G**) BLL (*p* < 0.01).

A total of 712 male workers were monitored, and sex hormones were recorded in 2019 and 2020. The multivariate analysis examining the association between variations in sex hormones and two-year blood Pb levels (BLLs) is presented in Table S2. This analysis was adjusted for confounding variables, including age, body mass index, smoking, and alcohol consumption. Both serum testosterone (β = 0.05, 95% CI: 0.005–0.09, *p* = 0.030) and luteinizing hormone (LH) (β = 0.27, 95% CI: −0.05–0.059, *p* = 0.093) demonstrated a positive association with BLLs. Figure 4 illustrates the levels of sex hormones and BLLs for Pb-exposed workers in 2019 and 2020. Paired-sample *t*-tests and Wilcoxon signed-rank tests were employed to evaluate the differences in hormone means and BLL means between the two years. The results indicated that testosterone (*p* < 0.001) and estradiol (E2) (*p* < 0.001) were significantly lower in 2020 than in 2019, while LH (*p* < 0.001), prolactin (PRL) (*p* < 0.001), and blood Pb (*p* < 0.001) levels were significantly higher in 2020 compared to 2019; further details are provided in Table S3. Pearson correlation analysis was performed to investigate the relationship between changes in sex hormones and changes in BLLs from 2019 to 2020, as detailed in Table S4.

### 3.4. Sex Hormone Changes in Patients Before and After Pb Chelation Therapy

**Figure 5:**
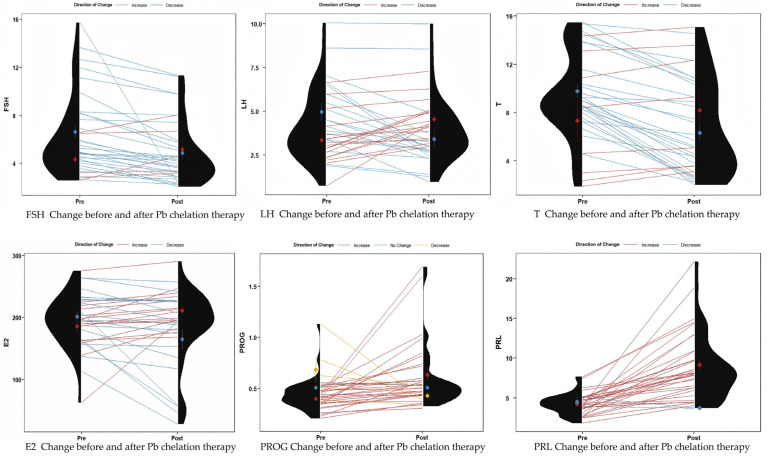
A violin chart showing the changes in hypothalamic and pituitary sex hormones in patients before and after Pb chelation therapy. FSH (*p* < 0.001); LH (*p* = 0.460); T (*p* < 0.001); E2 (*p* = 0.555); PROG (*p* < 0.001); PRL (*p* < 0.001).

A total of 35 male patients (mean age: 36.9 ± 5.7 years) undergoing Pb discharge treatment for Pb poisoning were evaluated. Figure 5 and Table 3 illustrate the changes in serum sex hormone levels before and after Pb chelation therapy. Following treatment, the mean blood Pb level (BLL) significantly decreased from 61.66 μg/dL to 36.30 μg/dL. Additionally, serum testosterone (T) levels decreased significantly from 9.21 ± 3.62 ng/mL to 6.75 ± 3.97 ng/mL (*p* < 0.001), and serum follicle-stimulating hormone (FSH) levels decreased from 6.32 ± 3.34 mIU/mL to 4.92 ± 2.59 mIU/mL (*p* = 0.002) compared to pre-chelation levels. These findings are consistent with the results of our cross-sectional study. Serum luteinizing hormone (LH) also showed a decrease from 4.19 ± 2.02 mIU/mL to 3.97 ± 1.91 mIU/mL (*p* = 0.460), although this change was not statistically significant. In contrast, serum prolactin (PRL) levels significantly increased from 4.25 ± 1.34 ng/mL to 8.85 ± 4.28 ng/mL (*p* < 0.001), and progesterone (PROG) levels also increased significantly from 0.44 ± 0.16 ng/mL to 0.60 ± 0.31 ng/mL (*p* = 0.011) after treatment.

## 4. Discussion

In recent years, the impact of heavy metals has emerged as a critical issue in public health, particularly concerning reproductive health, as abnormal levels of sex hormones have been linked to compromised semen quality. A recent report [24] determined that the levels of luteinizing hormone (LH), follicle-stimulating hormone (FSH), and testosterone (T) are all inversely associated with sperm motility. Furthermore, the levels of LH and FSH are also inversely associated with the rate of normal sperm morphology. However, the relationship between Pb exposure and male sex hormone levels remains controversial.

As a prevalent environmental pollutant, Pb possesses a complex and significant mechanism of toxicity within the central nervous system (CNS). The CNS is a primary target for Pb, which can inflict damage through several pathways, including the induction of oxidative stress [25], neuroinflammatory responses [23], the disruption of synaptic plasticity, interference with calcium signaling [26], and alterations in epigenetic regulation [27]. The CNS is also intricately linked to hormonal regulation. The hypothalamus, central to neuroendocrine regulation [28], orchestrates the release of various hormones via the pituitary portal system. It integrates information from other brain regions and the external environment, coordinating physiological processes such as metabolism, reproduction, and stress response [29]. Furthermore, it regulates the synergistic effects of the hypothalamic–pituitary–gonadal (HPG) axis [30]. There exists a bidirectional feedback mechanism between the CNS and hormones; the nervous system influences peripheral endocrine glands through the release of prohormone-releasing factors (e.g., GHRH), while hormones (e.g., estrogens and glucocorticoids) feed back into the CNS, impacting neuronal plasticity and neurotransmitter release [31,32]. Additionally, the hypothalamus plays a crucial role in regulating both energy metabolism and reproductive function, with leptin being implicated in CNS regulation. Additionally, the hypothalamus plays a crucial role in regulating both energy metabolism and reproductive function [33]. Metabolic hormones, such as leptin and ghrelin, interact with neurons in the arcuate nucleus of the brain, traversing the blood–brain barrier to establish a functional coupling with gonadotropin-releasing hormone neurons. Furthermore, the hypothalamic–pituitary–gonadal (HPG) axis operates as a dynamic equilibrium system; any abnormalities in these interactions may lead to dysfunctions within the HPG axis, resulting in disorders of sex hormone regulation.

Our study integrates findings from occupational epidemiological surveys and experimental epidemiology. Data from all Pb-exposed workers in cross-sectional studies and experimental investigations indicate a positive correlation between Pb exposure levels and serum testosterone levels. A gradual increase in serum testosterone levels was observed across groups with varying Pb exposure levels, with higher serum testosterone levels generally found in individuals with greater Pb exposure (Table 2, Figure 2A–C). In the cross-sectional analysis, serum luteinizing hormone (LH) levels demonstrated a significant association with both blood Pb levels (BLLs) and serum testosterone (T), suggesting a potential mediating role of LH in the relationship between BLLs and T (Figure 3). Notably, when BLLs exceeded 30.0 μg/dL, the relationship between the BLL and T appeared to be statistically explained by LH levels. In participants with BLLs above 30.0 μg/dL, the association between the BLL and T was fully attenuated after adjusting for LH levels, indicating that pituitary-derived hormones may predominantly mediate the effects of high-level Pb exposure on male reproductive hormones. Additionally, elevated serum follicle-stimulating hormone (FSH) levels were observed in individuals with higher BLLs (Figure 2). Collectively, these patterns support the hypothesis that Pb exposure may disrupt the hypothalamic–pituitary–gonadal (HPG) axis, potentially altering the regulatory pathways of T, FSH, and LH. However, due to the cross-sectional design of this study, it only suggests the existence of one potential pathway of association that warrants further exploration in longitudinal or experimental studies.

Our data demonstrate a similar trend in the nonlinear relationship between blood Pb levels (BLLs) and follicle-stimulating hormone (FSH) and luteinizing hormone (LH). Specifically, when the BLL is less than 20.5 μg/dL or greater than 30.0 μg/dL, both hormones exhibit an increase with a rising BLL (Figure 2A,B). Furthermore, when the BLL exceeds 28.09 μg/dL, testosterone (T) levels show a positive linear correlation with an increasing BLL. The results of our epidemiological trial involving patients undergoing Pb chelation therapy corroborate these associations (see Table 3) and are consistent with previous reports [10,20,34]. Table S1 indicates that LH levels were significantly elevated in the highest blood Pb level (BLL) group (≥40 μg/dL) compared to the lowest BLL group (<10 μg/dL). This observation aligns with findings from a previous study [35], which reported that groups with higher BLL values exhibited elevated LH concentrations relative to groups with mean BLL values below 10 μg/dL. Moreover, these results are consistent with our findings from the restricted cubic spline (RCS) model in the low dose range.

A recent review of epidemiologic studies reported no association between sex hormones and blood Pb levels (BLLs) [18]. However, the levels of follicle-stimulating hormone (FSH) and luteinizing hormone (LH) in our study differed from those in some previous reports. Our study demonstrated a nonlinear dose–response relationship between BLLs and both LH and FSH, which was not significantly correlated with linear regression. This discrepancy may further elucidate why such associations have not been observed in most prior epidemiologic studies. Factors contributing to the nonlinear relationship between BLLs and sex hormones may include the presence of a Pb toxicity threshold, the body’s gradual adaptation to Pb exposure, and the phenomenon of ‘regression to the mean’ of sex hormones. A notable distinction between our study and others is that our sample size was considerably larger than that reported in the review ([18]; the largest sample size reported was 341 male workers). Furthermore, we utilized the restricted cubic spline (RCS) model to explore the dose–response relationship between BLLs and sex hormone levels.

Changes in serum hormone levels following Pb exposure suggest a potential disruption of the hypothalamic–pituitary–thyroid (HPT) axis, which may consequently impair reproductive physiology and behavior. Consistent with our findings, the hypothalamus has been identified as an organ that accumulates Pb [36]. Previous animal studies have demonstrated that Pb exposure leads to alterations in gonadotropin-releasing hormone (GnRH) levels [37]. Furthermore, pituitary cells derived from Pb-treated animals exhibited a significant increase in luteinizing hormone (LH) release compared to control animals [37]. The observed elevation in LH and follicle-stimulating hormone (FSH) levels following Pb exposure may be attributed to increased GnRH levels in the hypothalamus. A prior study [38] noted a significant dose-dependent increase in GnRH mRNA concentration associated with escalating Pb doses in animals subjected to treatment for one week. Therefore, Pb exposure may adversely impact male reproductive health by affecting the hypothalamus, thereby triggering hormonal disturbances in pituitary and gonadal secretions, which disrupt the hypothalamic–pituitary–gonadal axis and lead to subsequent alterations in male sex hormone levels (A detailed comparison with existing relevant studies is shown in Table 4).

To the best of our knowledge, this study explores the effects of Pb on sex hormones in the largest sample of workers exposed to Pb-dominated environments, involving a total of six hormones. Additionally, we applied restricted cubic spline (RCS) modeling for the first time to estimate a nonlinear dose–response relationship between Pb exposure and sex hormone levels. Unlike previous environmental studies analyzing co-exposure to mixed metals, this occupational investigation focused on the main effects of Pb exposure at dose levels well above those typically encountered in non-occupational populations (however, potential co-exposure of other metals at background levels is unavoidable). Our findings indicate that subjects with higher Pb levels are more significantly affected by Pb compared to other studies in which Pb exposure was dominant or involved Pb and mixed-metal exposure. Furthermore, we synthesized results from epidemiologic investigations and treatment trials. However, our study has several limitations. First, due to its cross-sectional design, it is not possible to determine the temporal order and causality between the variables; thus, the results of this study reflect only statistical correlations. The mediation analysis presented is merely an exploratory hypothetical model that requires further verification in future experimental studies.

## 5. Conclusions

Our cross-sectional analysis of occupational epidemiology and experimental observations demonstrates a link between Pb exposure and altered sex hormone profiles in men. Specifically, we observed a nonlinear dose–response relationship between Pb exposure and serum levels of luteinizing hormone (LH) and follicle-stimulating hormone (FSH) in men. Furthermore, occupational epidemiological studies have indicated that Pb exposure increases serum testosterone (T) levels. In a cross-sectional study, mediation analysis suggests a potential role of LH in linking BLLs to T alterations in high-exposure groups. Finally, epidemiological trials have demonstrated that Pb chelation therapy partially restores hormone levels.

## Figures and Tables

**Table 1:** Characteristics of the study population.

Groups	Mean ± SD/No. (%)		
	Cross-Sectional Study (N = 1573)	Repeated Cross-Sectional Study from 2019 to 2020 (N = 712)	Pb Chelation Therapy Experiment (N = 35)
Age (years) ^a^	36.8 ± 7.2	36.4 ± 7.1	36.9 ± 5.7
BMI (kg/m^2^) ^a^	24.43 ± 3.56	24.44 ± 3.46	23.86 ± 2.80
Smoking status ^b^			
Never smoker	637 (41)	298(42)	15 (43)
Smoker	928 (59)	411 (58)	20 (57)
Alcohol drinking ^b^			
Never drinker	699 (44)	301 (43)	14 (40)
Alcohol user	842 (54)	397 (57)	21 (60)
Blood Pb (μg/dL) ^a^	24.96 ± 11.47	24.62 ± 11.86	61.66 ± 6.32
Pituitary hormones ^a^			
FSH (mlU/mL)	6.15 ± 3.47	6.16 ± 3.21	6.32 ± 3.34
LH (mlU/mL)	4.16 ± 1.98	4.21 ± 2.31	4.19 ± 2.12
Prolactin (ng/mL)	5.78 ± 4.29	5.80 ± 3.56	4.25 ± 1.34
Testis hormones ^a^			
Estradiol (pg/mL)	72.91 ± 36.01	73.23 ± 35.66	194.29 ± 43.74
Testosterone (ng/mL)	4.58 ± 1.99	4.61 ± 2.02	9.21 ± 3.62
Renicapsule hormones ^a^			
Progesterone (ng/mL)	0.53 ± 0.17	0.54 ± 0.22	0.44 ± 0.16

^a^: The results are presented as the mean ± SD. ^b^: The results are presented as the No. (%). FSH: follicle-stimulating hormone; LH: luteinizing hormone; BMI = body mass index.

**Table 2:** Association between blood Pb and sex hormones in different blood Pb levels (N = 1573).

	FSH	LH	E2	T	PROG
Low-BLL group (N)	616	616	236	421	659
Concentration (μg/dL)	<20.5	<20.5	≤13.0	<16.89	≤21.26
Model I					
β (95%CI)	0.069 (0.002, 0.137)	0.030 (−0.006, 0.067)	−0.402 (−2.023, 1.219)	0.034 (−0.019, 0.086)	−0.002 (−0.006, 0.001)
*t* value	2.03	1.62	−0.49	1.25	−1.37
*p* value	0.043	0.105	0.626	0.213	0.171
Model II					
β (95%CI)	0.069 (0.002, 0.137)	0.030 (−0.007, 0.067)	−0.179 (−1.8863, 1.504)	0.005 (−0.048, 0.057)	−0.003 (−0.007, 0)
*t* value	2.02	1.57	−0.21	0.17	−1.82
*p* value	0.044	0.118	0.834	0.862	0.069
Middle-level group (N)	468	468	-	590	-
Dose (μg/dL)	20.5–30.0	20.5–30.0	-	16.89–28.09	-
Model I					
β (95%CI)	−0.041 (−0.159, 0.072)	0.011 (−0.057, 0.079)		0.007 (−0.044, 0.059)	
*t* value	−0.75	0.31		0.28	
*p* value	0.456	0.753		0.779	
Model II					
β (95%CI)	−0.017 (−0.130, 0.096)	0.019 (−0.049, 0.086)		−0.008 (−0.059, 0.043)	
*t* value	−0.30	0.54		−0.31	
*p* value	0.765	0.588		0.755	
High-BLL group (N)	489	489	1337	562	914
Dose (μg/dL)	>30.0	>30.0	>13.0	>28.09	>21.26
Model I					
β (95%CI)	0.020 (−0.019, 0.060)	0.028 (0.005, 0.052)	0.155 (−0.037, 0.347)	0.023 (0.002, 0.045)	0.001 (0, 0.002)
*t* value	1.00	2.35	1.58	2.12	1.09
*p* value	0.318	0.019	0.113	0.035	0.278
Model II					
β (95%CI)	0.019 (−0.021, 0.058)	0.027 (0.003, 0.051)	0.152 (−0.043, 0.348)	0.027 (0.005, 0.049)	0.001 (0, 0.002)
*t* value	0.92	2.20	1.53	2.41	1.18
*p* value	0.359	0.028	0.127	0.016	0.240

Model I indicated the results of linear regression without adjusting other factors, and Model II indicated the results of multilinear regression adjusted for age, body mass index, smoking, and alcohol consumption. FSH: follicle-stimulating hormone; LH: luteinizing hormone; E2: estrogen; T: testosterone; PROG: progesterone.

**Table 3:** Changes in sex hormones in patients before and after Pb chelation therapy (N = 35).

	Before Pb Chelation Therapy		After Pb Chelation Therapy		*t* ^ a^	*p* ^ a^	*Z* ^ b^	*p* ^ b^
	Mean ± SD	P50 (P25, P75)	Mean ± SD	P50 (P25, P75)				
BLL (μg/dL)	61.66 ± 6.32	61.13 (58.59, 64.75)	36.30 ± 5.52	35.47 (32.39, 39.15)	25.86	<0.001	−5.16	<0.001
FSH (mlU/mL)	6.32 ± 3.34	4.95 (4.09, 8.04)	4.92 ± 2.59	4.06 (3.01, 5.87)	3.45	0.002	−4.14	<0.001
LH (mlU/mL)	4.19 ± 2.02	3.70 (2.76, 5.85)	3.97 ± 1.91	3.61 (2.77, 4.92)	0.75	0.460	−0.52	0.6000
T (ng/mL)	9.21 ± 3.62	8.77 (7.32, 11.39)	6.75 ± 3.97	5.17 (3.22, 10.08)	6.19	<0.001	−4.32	<0.001
E2 (pg/mL)	194.30 ± 43.74	196.86 (163.54, 224.63)	186.14 ± 60.94	195.90 (174.09, 227.00)	0.97	0.336	−0.59	0.555
PROG (ng/mL)	0.44 ± 0.16	0.41 (0.34, 0.51)	0.60 ± 0.31	0.51 (0.42, 0.59)	−2.68	0.011	−3.58	<0.001
PRL (ng/mL)	4.25 ± 1.34	4.42 (3.58, 5.04)	8.85 ± 4.28	8.10 (6.01, 10.92)	−6.39	<0.001	−5.06	<0.001

^a^: Paired sample *t*-test was applied. ^b^: Mann–Whitney Test was used. FSH: follicle-stimulating hormone; LH: luteinizing hormone; E2: estrogen; T: testosterone; PROG: progesterone; PRL: prolactin.

**Table 4:** Comparative table of the literature cited in the Discussion section.

Reference	Study Type	Sample Characteristics	Blood Pb Level (BLL)	Key Findings
This Study	Cross-sectional study	1753 male Pb-exposed workers	Mean: 24.96 μg/dL; median: 23.63 μg/dL	Positive correlation between Pb exposure level and testosterone (T) levels; LH levels significantly linked to BLL and serum T
	Repeated cross-sectional study	712 male Pb-exposed workers	2019 mean: 19.75 μg/dL; 2020 mean: 24.62 μg/dL	BLL, LH, and PRL were significantly higher in 2020 compared to 2019, while T and E2 were significantly lower in 2019
	Clinical study	35 male workers with occupational Pb poisoning	BLL decreased from 61.66 μg/dL to 36.30 μg/dL	Significant decline in serum T and FSH; no significant change in LH
[24]	Cross-sectional study	338 men from infertile couples	/	LH, FSH, and T levels inversely correlated with sperm motility
[10]	Cross-sectional study	98 subjects with slight to moderate occupational exposure to Pb and 51 reference subjects	Control group mean: 109 μg/dL; Pb-exposed workers mean: 387 μg/dL	Increased serum T and E2 in Pb-exposed group
[20]	Cross-sectional study	869 non-hospitalized civilians	Median BLL: 2.0 μg/dL	Positive correlation between BLL and T
[34]	Cross-sectional study	One hundred and two infertile men with occupational exposure and thirty fertile men.	Exposed group median: 43.80 μg/dL; control group median: 24.50 μg/dL	Higher FSH and lower T in heavy metal-exposed group compared to controls
[35]	Cross-sectional study	225 school-aged children (113 boys) near a ferromanganese plant	Boys’ median BLL: 1.20 μg/dL	Testosterone and LH concentrations were statistically higher in boys with an increased BLL
[18]	Systematic review and meta-analysis	26 studies on occupational exposure to heavy metals (e.g., Cd, Cr, Se, and Zn)	Significantly higher BLL in Pb-exposed groups vs. controls	Pb-exposed males showed comparable testosterone, FSH, and LH levels to unexposed controls
[36]	Animal study	32 male mice	/	Hypothalamus identified as a Pb accumulation site
[37]	In vivo and in vitro experiments	36 Pb-exposed male rats; 6 controls	The blood Pb levels of Pb-dosed animals were significantly higher than control animals in all experiments	Pb exposure caused abnormal GnRH levels and significant LH elevation
[38]	Animal study	Pb-exposed male rats	Control group: <3 µg/mL; Pb-exposed group: higher than controls	The signals within and between the hypothalamus and pituitary gland appear to be disrupted by long-term, low-dose Pb exposure

## Data Availability

The data used to support the findings of this study are available from the corresponding author upon reasonable request.

## Supplementary Materials

The following supporting information can be downloaded at https://www.mdpi.com/article/10.3390/toxics13050415/s1, Table S1: Effect of blood Pb on sex hormones in male Pb-exposed workers (N = 1573); Table S2: Multivariate analysis of the relationship between the blood Pb difference and sex hormone difference between 2019 and 2020 in a longitudinal study (N = 712); Table S3: The sex hormone levels in the Pb-exposed workers followed up both in 2019 and 2020 (N = 712); Table S4: Correlation analysis of blood Pb difference and sex hormone difference between 2019 and 2020 in male Pb-exposed workers (N = 712).
