# Supplementary material for: Effects of Lead Exposure on 1573 Male Workers’ Sex Hormones in China
Source: Toxics. 2025 May 21;13(5):415. doi: 10.3390/toxics13050415 (PMC12115724; doi:10.3390/toxics13050415)
Supplement: Supplementary file 1 [file toxics-13-00415-s001.zip › Informed Consent.pdf]

Informed Consent Form

Title of Study: An Investigative Study on Male Reproductive Health and Factors Affecting It (Grant No. 2017YFC1002001)

Project Leader: Xinxiang Medical University, School of Public Health; Dr. Zhang Guanghui

Contact Information: Tel: 0373-3831027 / 18237376831

1. Study Overview and Purpose

There has been a documented decline in semen quality among males over the past century. Environmental pollution, societal stress, and psychological factors are among the potential contributors to this trend. This study aims to investigate semen quality in adult males and its associated risk factors. Participants will:

Complete a questionnaire under the supervision of a licensed hospital physician.

Provide semen, blood, and urine samples for analysis, including blood lead levels, sex hormone testing, and genetic/epigenetic studies.

Receive a personalized health evaluation from a physician, including insights into reproductive health.

2. Participation Agreement

By agreeing to participate, you will sign this document to confirm your voluntary consent after reviewing all study details.

3. Study Procedures

Questionnaire: A 10-minute survey covering demographics, lifestyle (e.g., smoking, alcohol use), and psychological factors.

Physical Measurements: Height, weight, and other basic health metrics.

Sample Collection: Peripheral blood, urine, and semen specimens will be collected under sterile, standardized conditions.

4. Potential Risks

Privacy Risks: All participants will sign a confidentiality agreement to ensure personal data is securely handled and not disclosed.

Blood Collection: Procedures will adhere to strict safety protocols to minimize discomfort or infection risks.

5. Benefits of Participation

Receive a comprehensive health assessment, including early detection of potential health issues.

Contribute to advancing scientific understanding of male reproductive health.

6. Confidentiality Assurance

All data and samples will be anonymized and accessible only to authorized researchers. Records will be stored securely and destroyed after study completion.

7. Participant Rights

You may withdraw from the study at any time without penalty or impact on your medical care.

For concerns or complaints, contact the Ethics Committee of Xinxiang Medical University at 0373-3831589.

Participant Signature: ______________________________ Date: ____________

Investigator Signature: _____________________________ Date: ____________
