# Supplementary material for: Effects of Lead Exposure on 1573 Male Workers’ Sex Hormones in China
Source: Toxics. 2025 May 21;13(5):415. doi: 10.3390/toxics13050415 (PMC12115724; doi:10.3390/toxics13050415)
Supplement: Supplementary file 1 [file toxics-13-00415-s001.zip › Tables S.pdf]

Table S 1. Effect of blood lead on sex hormones in male lead exposed workers (N=1573).

**Blood lead (μg/dL)**	**FSH (mlU/ml)**	**LH (mlU/ml)**	**T (ng/ml)**	**E2 (pg/ml)**	**PROG (ng/ml)**	**PRL (ng/ml)**
<10 (116)	5.88 ± 3.46	3.85 ± 1.72	**4.28 ± 1.71**	74.69 ± 32.92	0.51 ± 0.15	5.98 ± 3.94
10~ (471)	6.25 ± 3.70	4.18 ± 2.03	**4.57 ± 2.03**	70.48 ± 34.32	0.54 ± 0.21	5.86 ± 3.58
20~ (497)	6.18 ± 3.36	4.22 ± 1.98	**4.52 ± 2.00**	73.11 ± 37.19	0.53 ± 0.15	5.81 ± 4.68
30~ (331)	5.97 ± 3.11	4.00 ± 1.86	**4.60 ± 1.91**	73.27 ± 36.59	0.53 ± 0.13	5.68 ± 4.99
≥40 (158)	6.31 ± 3.81	**4.43 ± 2.22***	**4.95 ± 2.26****	77.49 ± 37.90	0.55 ± 0.13	5.46 ± 3.52
P^a	0.676	0.078	**0.073**	0.286	0.443	0.825
P^b	0.960	0.282	**0.021**	0.144	0.315	0.240
$\chi^{2\,c}$	2.03	7.45	5.85	5.37	4.08	**9.33**
P^c	0.731	0.114	0.211	0.252	0.395	**0.053**

a ANOVA test was applied. P^a indicates P value of ANOVA, and P^b indicates P value for trend. c Kruskal-Wallis test was used.

"*" indicates $P<0.05$, "**" indicates $P<0.01$ gotten from LSD method.

FSH: follicle-stimulating hormone; LH: luteinizing hormone; E2: estrogen; T: testosterone; PROG: progesterone and PRL: prolactin

Table S 2. Multivariate analysis of the relationship between the blood lead difference and sex hormone difference between 2019 and 2020 in longitudinal study (N=712).

**Characteristic**	**δFSH**	**δLH**	**δT**	**δE2**	**δPROG**	**δPRL**
Model Ⅰ						
β (95%CI)	-0.003(-0.03,0.02)	**0.29(-0.03,0.60)**	**0.04(-0.005,0.08)**	0(-0.06,0.06)	-0.002(-0.02,0.01)	0.01(-0.01,0.02)
Wald χ^2	0.04	**3.10**	**3.02**	0	0.14	0.99
P value	0.837	**0.078**	**0.082**	0.995	0.713	0.320
Model Ⅱ						
β (95%CI)	-0.005(-0.03,0.02)	**0.27(-0.05,0.59)**	**0.05(0.005,0.09)**	0.002(-0.06,0.06)	-0.001(-0.01,0.01)	0.01(-0.01,0.02)
Wald χ^2	0.13	**2.83**	**4.71**	0.01	0.04	0.97
P value	0.717	**0.093**	**0.030**	0.944	0.837	0.325

Model Ⅰ indicated the results of generalized linear models for Ln blood lead difference between 2019 and 2020. Model Ⅱ indicated the results of generalized linear models adjusted for Ln blood lead difference, after adjusting age, body mass index, smoking, and alcohol consumption.

FSH: follicle-stimulating hormone; LH: luteinizing hormone; E2: estrogen; T: testosterone; PROG: progesterone and PRL: prolactin.

Table S 3. The sex hormones levels in the lead exposed workers followed-up both in 2019 and 2020 (N=712).

	**2019**	**2020**	t^a	P^a	Z^b	P^b

	Mean±SD	P50(P25, P75)	Mean±SD	P50(P25, P75)				
FSH (mlU/ml)	5.86 ± 3.02	5.20 (3.78,7.29)	6.16 ± 3.21	5.38 (3.89,7.62)	1.16	0.246	1.27	0.204
LH (mlU/ml)	3.58±1.98	3.30 (2.43,4.32)	4.21 ± 2.31	3.82 (2.87,5.18)	**5.47**	**<0.001**	**6.65**	**<0.001**
T (ng/ml)	5.81 ± 3.06	5.25 (3.35,7.87)	4.61 ± 2.02	4.22 (3.12,5.96)	**8.69**	**<0.001**	**7.00**	**<0.001**
E2 (pg/ml)	104.13 ± 59.91	91.35(65.30,131.05)	73.23 ± 35.66	65.57(45.02,93.92)	**11.83**	**<0.001**	**11.51**	**<0.001**
PROG (ng/ml)	0.87 ± 2.98	0.53 (0.42,0.64)	0.54 ± 0.22	0.53 (0.45,0.61)	2.61	0.009	0.41	0.686
PRL (ng/ml)	4.81± 4.61	3.97 (2.99,5.35)	5.80 ± 3.56	4.95 (3.81,6.61)	**2.69**	**<0.007**	**9.60**	**<0.001**
Blood lead (μg/dL)	19.75 ± 8.27	19.40 (12.54,26.68)	24.62 ± 11.86	23.07(15.35,32.12)	**8.82**	**<0.001**	**7.21**	**<0.001**

a Paired sample t test was applied.

b Mann-Whitney Test was used.

FSH: follicle-stimulating hormone; LH: luteinizing hormone; E2: estrogen; T: testosterone; PROG: progesterone and PRL: prolactin.

Table S 4. Correlation analysis of blood lead difference and sex hormone difference between 2019 and 2020 in male lead exposed workers (N=712).

Correlation	δFSH	**δLH**	δTesto	δE2	**δPROG**	δPRL
δPb						
Correlation coefficient	-0.064	**0.055**	0.059	0.033	**-0.006**	-0.047
P	0.074	**0.127**	0.103	0.368	**0.875**	0.193

The correlation of serum hormones difference and blood lead difference between 2019 and 2020 was analyzed by Pearson correlation.

FSH: follicle-stimulating hormone; LH: luteinizing hormone; E2: estrogen; T: testosterone; PROG: progesterone and PRL: prolactin.
